# Evaluation of the Chinese version of the constipation scoring system in Chinese women with pelvic organ prolapse

**DOI:** 10.1038/s41598-022-11312-7

**Published:** 2022-05-05

**Authors:** Yanhua Liu, Man Tan, Cheng Tan, Xin Yang

**Affiliations:** 1 Department of Gynaecology, Peking University People’s Hospital, 11 Xizhimen South Street, Xicheng District, Beijing, China; 2 Department of Gynecological Pelvic Floor and Oncology, Chongqing Health Center for Women and Children, Chongqing, China

**Keywords:** Urogenital diseases, Diagnosis

## Abstract

Defecation disorder is one of the main symptoms in pelvic organ prolapse (POP) patients. Our study aims to translate the Chinese version of the constipation scoring system (CSS) questionnaire and test the reliability and validity of its application in Chinese POP women. We recruited 140 women suffering prolapse with stage II or above POP who reported at least one abnormal defecation symptom. We chose CRADI-8 as the criterion validity. Cronbach's α coefficient of the total score of CSS was 0.721, the ICC and Kappa coefficient of CSS total score were 0.877 and 0.424, respectively. The total score of CSS was significantly correlated with the CRADI-8 score (p < 0.001), and Spearman's coefficient was 0.491. The total score of CSS in the women with constipation (according to Rome III criteria) was greater than that of patients without constipation (p < 0.001), and the total score of CSS was significantly different before and after surgery (p < 0.001), which was consistent with the change of CRADI-8. The median of the preoperative group was 6(3,10), and the median of the postoperative group was 3(0,7). These data demonstrate that the Chinese version of the CSS questionnaire has great internal consistency, retest reliability, and construct validity. It may be widely used to evaluate constipation symptoms in women with pelvic organ prolapse in China.

## Introduction

Pelvic organ prolapse (POP) is a common disease among older women, leading to various symptoms^1^. The prevalence rate of posterior pelvic prolapse is 18–40%^2^. The severity of posterior pelvic prolapse is closely related to defecation disorders^3^. Constipation was a rising concern of POP patients. Obstructed defecation affected as much as 43% of patients with pelvic organ prolapse^4^.

With increasing attention to pelvic floor disorders, a series of validated self-administered QOL questionnaires have been developed to assess individual symptoms^5^. However, the validated Chinese version of questionnaires inquiring into functional constipation was limited. Although the Rome III criterion for functional constipation is considered the golden standard, it is not possible to assess the severity of the symptoms of constipation^6^. Pelvic Floor Impact Questionnaire Short Form 7 (PFIQ-7) and Pelvic Floor Distress Inventory 20 (PFDI-20) were developed in 2005^7^. The Chinese translation version of the questionnaires and their reliability and validity analysis among the Chinese patients were completed in 2011 and 2019, respectively^8,9^. They were widely used among patients with pelvic organ prolapse. However, PFIQ-7 and PFDI-20 were not designed for investigating symptoms of constipation^7^.

The Constipation Scoring System (CSS), also known as Wexner Constipation Scores or Cleveland Constipation Scores, is one of the earliest and most widely accepted Constipation Scores. The original English version of the Constipation Scoring System was developed and verified by the Colorectal Surgery Department, Cleveland Clinic, USA^10^. CSS is widely used to assess the severity of constipation in China^11^. However, no Chinese version of CSS has been analyzed for reliability and validity, nor is there verification research on the Chinese POP women. As pelvic floor gynecologists increasingly pay attention to assessing posterior pelvic symptoms, CSS has shown excellent prospects in applying to POP patients. The use of CSS in the Chinese population may have cultural or linguistic issues. Some items may need to be adjusted according to the characteristics of the POP patients to improve its understandability. The demand for translating CSS questionnaire into Chinese version and testing its validation in patients with POP has become increasingly urgent.

This study aims to translate the CSS questionnaire into Chinese and analyze its reliability and validity in POP patients, fill the blanks in the questionnaire for constipation assessment of POP patients, and promote the application of CSS in clinical work and research. In POP patients, it may provide a basis for establishing a comprehensive evaluation system for the anatomy and function of posterior pelvic compartment prolapse.

## Methods

It was designed as a prospective study approved by The Ethics Committee of Peking University People's Hospital (Ethics Number: 2019PHB273-01). The patients with pelvic organ prolapse were recruited from outpatient at Peking University People's Hospital from May 2019 to January 2021. All research was performed following relevant guidelines and regulations, including the Declaration of Helsinki.

Inclusion criteria: (1) Patients diagnosed with stage II or above pelvic organ prolapse according to POP-Q with ages ranging from 30 to 90; (2) Patients who report at least one abnormal defecation symptom; (3) Willing to accept relevant questionnaire survey.

Exclusion criteria: (1) Previous surgical treatment for pelvic organ prolapse; (2) Constipation was diagnosed as "colon slow transit constipation"; (3) Previous diagnosis of ulcerative colitis, Crohn's disease, or colorectal malignancy; (4) Unable to understand or complete the questionnaires; (5) Complicated with serious other somatic diseases (such as diabetes with unstable blood glucose levels, heart disease and malignant tumors in other body parts).

The CSS included eight questions: frequency of bowel movements, difficulty in evacuation effort, incomplete evacuation, abdominal pain, time in lavatory per attempt, type of assistance, unsuccessful attempts for evacuation per 24 h, and duration of constipation. The respondents evaluated the eight questions, and each question was scored according to the corresponding options (0–2 points for type of assistance, 0–4 points for other questions, and 30 points in total). The total score of the eight questions was the CSS total score. The larger the score of the questionnaire, the more serious the symptoms of constipation.

The Chinese Version of CSS was translated according to the "WHO-QOL Questionnaire Translation Method for Intercultural Quality of Life Research." We obtained the authorization of translation authentication and correlational research of the initial CSS questionnaire from the corresponding author via email. To maintain as much original meaning as possible, the CSS translation contains two dependent forward and backward translations^12^. We arranged a pilot study in 10 participants with POP-Q stage II or above. The official Chinese version of the CSS questionnaire was obtained according to the modified opinions of experts and the feedback of subjects. The Rome III criterion for functional constipation is an easy-to-use and straightforward method. The researchers diagnosed whether the patients met the Rome III criteria for functional constipation^6^. Patients were investigated with the Chinese version of the CSS questionnaire at a 2–4 weeks interval before the surgery by two separate researchers. The researcher of the second investigation was blinded from the database of the first investigation. The enrollment data are presented in Supplementary Fig. 1.

### Reliability analysis

Cronbach's α coefficient was used to evaluate the internal consistency of the questionnaire. Cronbach's α coefficient > 0.7 indicated that the internal consistency of the questionnaire was good. The intraclass correlation coefficient (ICC) was used to evaluate the retest reliability of the questionnaire. ICC > 0.75 indicated good retest reliability, 0.4 ≤ ICC ≤ 0.75 was medium, and ICC < 0.4 was poor. The Kappa coefficient was used for the consistency test of two measurements. The Kappa coefficient ≤ 0.21 was considered general consistency, and a Kappa coefficient ≤ 0.41 was considered medium consistency.

### Validity analysis

Factor analysis aims to determine whether the correlation between multiple observed variables can be explained or summarized by a smaller number of latent variables. Unobserved variables are also called factors^13^. There are two main methods: exploratory factor analysis and confirmed factor analysis. Exploratory factor analysis is used to conduct a preliminary investigation of a set of observed variables and confirmed factor analysis is a method used to test whether a specified factor structure is still valid for a new data set.

Construct validity: in exploratory factor analysis, we used the Kaiser–Meyer–Olkin test and Bartlett spherical test to determine whether the scale met the conditions of factor analysis. If the KMO value > 0.6 and p < 0.001 met the requirements of factor analysis, factor analysis could be carried out by principal component analysis to extract the common factors of feature root > 1 to judge whether the Chinese questionnaire had an excellent logical structure. In confirmed factor analysis, we use CMIN/DF, RMSEA, IFI, CFI, TLI, PNFI, PCFI to evaluate the model's fit, use AVE, CR to assess convergent validity and calculate the AVE arithmetic square root and correlation coefficient of the factors. If the arithmetic square root of AVE > correlation coefficient, it means that the discriminant validity is good.

Criterion validity: as a subscale of the PFDI-20 scale for the influence of defecation symptoms on quality of life, the CRADI-8 has been verified in the Chinese PFD population^9^. There is still no gold standard for evaluating defecation function in POP patients. Accordingly, the recommendations of consensus-based standards for the selection of health measurement instruments (COSMIN) were followed^14^. We chose the CRADI-8 as the criterion of efficacy. Spearman correlation coefficient analysis of the CSS score and CRADI-8 score was used to evaluate the validity of the criteria. If p < 0.05, the correlation between CSS score and CRADI-8 score is significant.

### Floor effect and ceiling effect

The floor effect and ceiling effect mean that if more than 15% of the respondents obtain the lowest or highest score, respectively, the floor effect and ceiling effect are considered to exist^15^.

### Reactivity

The Wilcoxon signed-rank test was used to assess whether there was a significant difference in CSS score before and after surgery. P < 0.05 indicated a significant difference between the two results.

### Statistical analysis

Excel was used to input data, and SPSS 24.0 statistical software (2016 version) and AMOS 21.0 were used for data analysis. SPSS 24.0 statistical software and GraphPad Prism 8.0.2 software were used for graphic drawing. Measurement data were expressed as the mean ± standard deviation (‾x ± s) or median (25th and 75th percentiles) (M (P25, P75)) according to data distribution characteristics, while classification data were expressed as frequency and percentage. Spearman correlation analysis was used to evaluate the correlation of measurement data between the two groups. The Mann–Whitney U test of two independent samples was used to compare independent measurement data between the two groups. P < 0.05 was considered statistically significant. Taking the Roman III criteria of functional constipation for reference, the curve of the receiver operating characteristic (ROC) curve of CSS in diagnosing constipation in POP patients was drawn. We calculated the area under the curve (AUC) and the optimal cut-off point to discuss the role of CSS in the diagnosis of constipation in POP patients.

### Ethical approval

The Ethics Committee of Peking University People's Hospital approved this study. (Ethics Number: 2019PHB273-01). All research was performed in accordance with relevant guidelines and regulations, including the Declaration of Helsinki. All the subjects were adults, and they obtained informed consent in the study.

## Results

### Demographic information and POP-Q staging of the participants

A total of 140 women were enrolled in our study and completed the Chinese version CSS questionnaire and CARDI-8 questionnaire. 90 of whom completed the second CSS questionnaire at a 2–4 weeks interval before the surgery, and 87 completed the postoperative follow-up, which was performed three months after surgery (See Supplementary Fig. 1). The demographic information is shown in Table 1.

**Table 1:** Demographic information of the participants.

	Minimum value	Maximum value	$\overline{{\rm x}} \pm {\rm s}$*	M (P_25_, P_75_)
**Enrolled Participants (n = 140)**				
Age (years)	33	86	63.65 ± 11.04	
Height (m)	1.48	1.72	1.60 ± 0.05	
Weight (kg)	43	84	62.89 ± 7.52	
BMI (kg/m^2^)	17.51	34.96	24.68 ± 2.89	
Number of pregnancies (times)	1	8		3 (2, 4)
Number of births (times)	1	6		2 (1, 3)
**Retest participants (n = 90)**				
Age (years)	35	86	66.20 ± 10.65	
Height (m)	1.50	1.72	1.60 ± 0.46	
Weight (kg)	43.0	84.0	63.55 ± 1.45	
BMI (kg/m^2^)	17.51	34.96	25.02 ± 3.13	
Number of pregnancies (times)	1	8		3 (2, 4)
Number of births (times)	1	6		2 (1, 3)

*$\overline{{\rm x}} \pm {\rm s}$ refers to the mean ± standard deviation, M (P_25_, P_75_) refers tothe median (25th percentile, 75th percentile).

### Reliability analysis

#### Internal consistency

The Cronbach's α coefficient of the total score of the CSS questionnaire in the Chinese version for POP patients was 0.721. The results of each item and the overall Cronbach's α coefficient if items were deleted are shown in Table 2. The overall Cronbach’s α coefficient only increased slightly (to 0.722) when deleting the fourth question, “How often do you suffer from abdominal pain?”. The above results indicate that the internal consistency of the questionnaire is excellent.

**Table 2:** Internal consistency of the CSS questionnaire in the Chinese version.

	Scope of score	Average	Standard deviation	Corrected item-total correlation	Cronbach’s α if item deleted
1. Frequency of bowel movements	0–4	0.18	0.420	0.394	0.709
2. Difficulty: painful evacuation effort	0–4	1.05	1.189	0.572	0.653
3. Completeness: feeling incomplete evacuation	0–4	1.36	1.247	0.429	0.694
4. Pain: abdominal pain	0–4	0.44	0.867	0.253	0.722
5. Time: minutes in lavatory per attempt	0–4	0.69	0.804	0.491	0.680
6. Assistance: type of assistance	0–2	0.55	0.834	0.374	0.700
7. Failure: unsuccessful attempts for evacuation per 24 h	0–4	0.56	0.552	0.472	0.694
8. History: duration of constipation (yr)	0–4	0.93	1.290	0.496	0.676

Except for the fourth item, pain, other items did not increase the overall Cronbach's α coefficient of the questionnaire after the deletion.

#### Test–retest reliability

For the 90 patients who completed the second investigation of CSS, the ICC analysis was conducted and shown in Table 3. The ICC of the total score of CSS was 0.877. The results showed that the retest reliability of the total score of the questionnaire was good, and the subitems were medium to good (0.690–0.920). The Kappa value of the total CSS score was 0.424, and the Kappa value of all questions ranged from 0.581 to 0.877. The results showed great consistency in the total score and the subitems.

**Table 3:** Retest reliability of the CSS questionnaire in the Chinese version.

	Mean ± standard deviation		Kappa value	ICC
	Test	Retest		
1. Defecate frequency	0.17 ± 0.40	0.18 ± 0.46	0.830	0.794
2. Difficulties: painful bowel movements	0.99 ± 1.26	0.82 ± 1.15	0.704	0.824
3. Empty: Incomplete emptying	1.57 ± 1.29	1.48 ± 1.27	0.595	0.695
4. Pain: abdominal pain	0.46 ± 0.89	0.31 ± 0.73	0.581	0.759
5. Time: Try defecation time (minutes)	0.87 ± 0.86	0.83 ± 0.88	0.814	0.875
6. Assisted defecation: Assisted type	0.74 ± 0.89	0.70 ± 0.88	0.787	0.774
7. Failure to defecate: The number of failed defecations per 24 h	0.69 ± 0.57	0.57 ± 0.58	0.675	0.690
8. History: Course of constipation (year)	1.22 ± 1.40	1.27 ± 1.40	0.877	0.920

Intraclass correlation analysis results showed that the ICCs of all items ranged from 0.690 to 0.920. The retest reliability of the questionnaire was good.

### Validity analysis

#### Construct validity

Factor analysis was performed on the CSS scores of 140 patients to verify the construct validity of the CSS questionnaire in the Chinese version. The Chinese CSS questionnaire's Kaiser–Meyer–Olkin test result was 0.807. Bartlett's test showed a very weak partial correlation (p < 0.001), indicating that this questionnaire is very suitable for factor analysis.

The result of factor analysis is shown in Table 4. Two components were extracted from the data (Eigenvalues > 1), which explained 36.293% and 13.058% of the total data variation, respectively. These two components accounted for 47.245% of the data variation after extraction. Table 5 shows the results of the rotated component matrix analysis. QuestionsQ1-Q5 and Q7 belonged to the first factor, and Q6 and Q8 belonged to the second factor. The variances for the contributions of all factors ranged from 52.0% to 88.0%. Although the original version did not test the construct validity, the results of the Chinese version show that the tool has excellent construct validity. Confirmed factor analysis was employed to assess construct validity from three aspects of model fit degree, convergent validity, and discriminative validity. As shown in Supplementary Table 1, all indicators are confirmed to indicate great model adaptation. The standard load coefficients of each indicator in the model are shown in Supplementary Table 2. The AVE of factor 1 is not greater than 0.4, indicating that the convergent validity is not ideal. The combined reliability CR is greater than 0.6, meaning that the basic fit of the model is good and has high structural validity. For discriminative validity, we calculated that the arithmetic square root of AVE of factor 1 is 0.530. The arithmetic square root of AVE of factor 2 is 0.705, both of which are greater than their correlation, 0.525, indicating that the discriminative validity of the two is good.

**Table 4:** Factor analysis of the CSS questionnaire in the Chinese version.

Component	Initial eigenvalues			Extraction sums of squared loadings		
	Total	% of the Variance	Cumulative %	Total	% of the variance	Cumulative %
1	2.662	33.272	33.272	2.662	33.272	33.272
2	1.118	13.973	47.245	1.118	13.973	47.245
3	0.941	11.762	59.007			
4	0.875	10.943	69.950			
5	0.0724	9.047	78.997			
6	0.623	7.786	86.783			
7	0.601	7.510	94.292			
8	0.457	5.708	100.000			

Extraction method: Principal Component Analysis. The first two main components of eigenvalues > 1were finally extracted.

**Table 5:** Rotated component matrix of the CSS questionnaire in the Chinese version.

	Factor	
	1	2
1. Defecate frequency	**0.660**	0.088
2. Difficulties: painful bowel movements	**0.704**	0.255
3. Empty: Incomplete emptying	**0.645**	−0.004
4. Pain: abdominal pain	**0.520**	−0.273
5. Time: Try defecation time (minutes)	**0.614**	0.066
6. Assisted defecation: Assisted type	−0.040	**0.880**
7. Failure to defecate: The number of failed defecations per 24 h	**0.532**	0.218
8. History: Course of constipation (year)	0.469	**0.555**

Q1-5 and Q7 belong to the first factor, and Q6 and Q8 belong to the second factor.

The bold means that this item has greater variance contribution of the factor.

#### Criterion validity

CRADI-8 is a questionnaire to assess the bothersomeness of defecation symptoms. The higher the score, the greater the annoying defecation symptoms in the patient^9^. The Spearman correlation coefficient between the total CSS score and the CRADI-8 in the Chinese version was r = 0.491, p < 0.001, which proved that the CSS score was significantly correlated with the CRADI-8 score.

### Hypothesis testing

We hypothesized that patients diagnosed with constipation according to the Roman III criteria for functional constipation had a higher overall CSS score than patients without constipation. Compared with patients without constipation according to Roma III criteria for functional constipation, patients with constipation had higher CSS scores (p < 0.001), the median of the patients without constipation group was 3(1,5), the median of the patients with constipation group was 8(5,12).

### Floor effect and ceiling effect

There were no patients with a CSS score of 30, the highest score was 21 (1/140, 0.7%), and the lowest was 0 (9/140, 6.4%). CSS application in Chinese POP women with defecation symptoms showed no significant floor effect or ceiling effect. The floor effect and ceiling effect of subitems are shown in Table 6. Only question 6, “type of assistance to evacuate,” showed ceiling effect. All questions showed floor effect.

**Table 6:** The floor/ceiling effect of different items in the CSS questionnaire.

	Ceiling effect	Floor effect
1. Defecate frequency	0	76(84.4%)
2. Difficulties: painful bowel movements	4(4.4%)	46(51.1%)
3. Empty: Incomplete emptying	7(7.8%)	23(25.6%)
4. Pain: abdominal pain	2(2.2%)	65(72.2%)
5. Time: Try defecation time (minutes)	1(1.1%)	35(38.9%)
6. Assisted defecation: Assisted type	27(30.0%)	50(55.6%)
7. Failure to defecate: The number of failed defecations per 24 h	0	33(36.7%)
8. History: Course of constipation (year)	10(11.1%)	40(44.4%)

Except for the assisted type, none of the items have ceiling effect. All items have floor effect.

### Reactivity

As for the 90 patients who completed the second CSS investigation, 87(96.7%) completed the telephone follow-up three months after surgery. The CSS and CRADI-8 scores are shown in Table 7. The median CSS score before and after surgery was 6(3,10) and 3(0,7), respectively, indicating a significant decrease after surgery, consistent with the change in the CRADI-8 score. This result showed that the CSS questionnaire in the Chinese version had excellent reactivity.

**Table 7:** Comparison of preoperative and postoperative CSS/CRADI-8 scores.

	Preoperative	Postoperative	p value
CRADI-8	4(2,7)	2(0,5.25)	0.000**
1. Hard bowel movement	1(0,2)	0(0,1)	0.004**
2. Empty: Incomplete emptying	1(0,2)	0(0,2)	0.484
3. Lose stool: well formed	0(0,0)	0(0,0)	0.747
4. Lose stool: loose	0(0,1)	0(0,0)	0.458
5. Lose gas	0(0,1)	0(0,0)	0.001**
6. Pain: pass stool	0(0,0)	0(0,0)	0.44
7. Urgency	0(0,2)	0(0,1)	0.041*
8. Outside	0(0,0)	0(0,0)	0.015*
CSS	6(3,10)	3(0,7)	0.000*
1. Defecate frequency	0(0,0)	0(0,0)	0.109
2. Difficulties: painful bowel movements	0(0,2)	0(0,0)	0.000**
3. Empty: Incomplete emptying	1(0,3)	1(0,2.25)	0.069
4. Pain: abdominal pain	0(0,1)	0(0,0)	0.028
5. Time: Try defecation time (minutes)	1(0,1)	0(0,1)	0.005**
6. Assisted defecation: Assisted type	0(0,2)	0(0,1)	0.015*
7. Failure to defecate	1(0,1)	0(0,1)	0.006**
8. History: Course of constipation (year)	1(0,2)	0(0,2)	0.253

The test results showed a significant difference in CSS and CRADI-8 scores before and after surgery (p < 0.001).

### Preliminary application of CSS in assessing posterior pelvic prolapse

**Figure 1:**
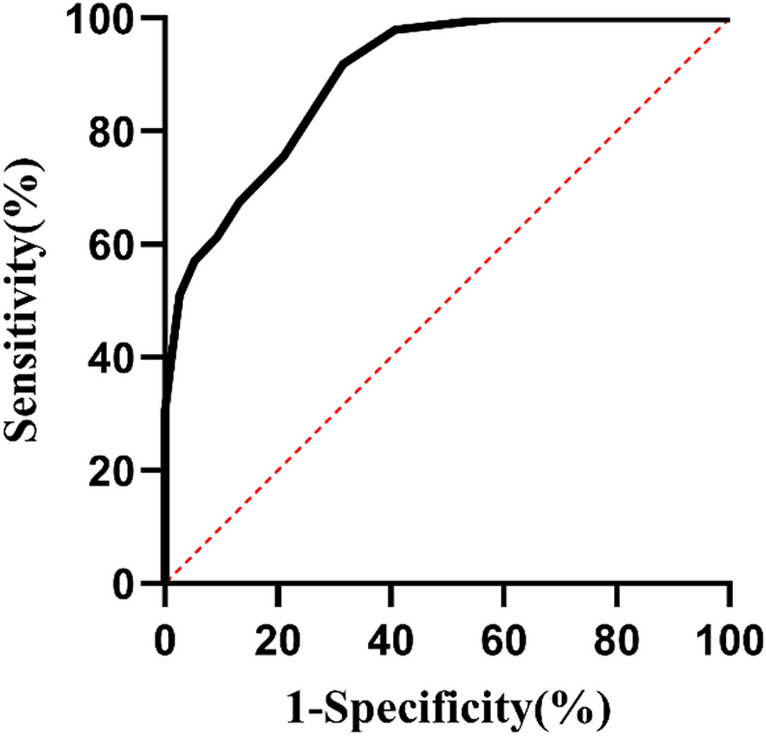
ROC curve of CSS score in the diagnosis of constipation.

The data from 125 patients with stage II or above posterior pelvic prolapse according to POP-Q was used to build the ROC curve using the Rome III criteria as reference (See Fig. 1). The AUC was 0.896 (95% CI 0.844–0.948), and the cut-off value was 4, providing a sensitivity of 91.84% and a specificity of 68.42%. The positive predictive value was 65.2%, and the negative predictive value was 92.9%.

## Discussion

The CSS questionnaire, also known as the Wexner questionnaire, is a common tool for examining the severity of constipation in research and the clinic, which takes less than 5 min to answer all questions^16^. In this study, we evaluated the reliability and validity of the Chinese version of the CSS questionnaire. The results showed good internal consistency, excellent test–retest reliability, good structural validity, and good criterion validity. All assumptions were confirmed. Thus, the translated version can be used as a standard tool in clinics and research in Chinese-speaking women with pelvic organ prolapse.

Although the original version of the CSS questionnaire is widely used, it has not been formally validated^17^. However, the Persian version of the CSS questionnaire has been proved to have good validity and reliability^16^. The objective of this study was to translate the CSS questionnaire to Chinese and test its reliability and validity among women with POP.

The internal consistency of the Chinese version was good, indicating that all items presented the severity of constipation. The good test–retest reliability guaranteed that the questionnaire results were consistent over time.

As for the construct validity, two components were found in factor analysis. The first factor consisted of Q1-Q5 and Q7, which might refer to the severity of constipation. The second factor consisted of Q6 and Q8. According to our unpublished data, the result of the balloon expulsion test was significantly associated with the type of assistance and duration of defecation. These results indicated there might be an internal connection between these two items. This model was confirmed to have good adaptation, discriminative validity, and moderate convergent validity.

The Chinese version of PFDI-20 has been validated and widely used in Chinese-speaking patients with POP^9^. For this reason, CARDI-8, the third domain of PFDI-20, was chosen to be the reference in criterion validity testing. The CSS score was significantly correlated with the score of CARDI-8, indicating a good sensitivity in evaluating the bothersomeness of defecation symptoms. The CSS score was significantly decreased after surgery, consistent with the change in the CRADI-8 score, indicating good reactivity of the CSS questionnaire.

The total score of the Chinese version of CSS among patients with POP had no obvious floor effect or ceiling effect. However, when it came to subitems, we found that all items had floor effects, especially defecation frequency, abdominal pain, and difficulties. There is no analysis of the floor effect in the original English questionnaire, so it is hard to compare with^8^. This might be because the degree of constipation among the selected POP women was not as severe as other studies which investigated patients with functional constipation^16^. This finding was in line with our former study^4^. The CSS questionnaire might be adjusted to be suitable for patients with POP in the future.

We also found that the CSS questionnaire had certain diagnostic value of functional constipation among patients with posterior vaginal prolapse. Based on the Roman III criteria of functional constipation, the AUC of CSS for the diagnosis of functional constipation was 0.896 (95% CI 0.844–0.948), the optimal cut-off value was 4. The CSS questionnaire was suitable to be used as an exclusion test for its negative predictive value was as high as 92.9%.

There are some strengths in this study. It is the first study to test the reliability and validity of the Chinese version of CSS among POP patients, providing a new reliable method for accessing the severity of defecation symptoms in Chinese POP women. Also, the prospective design and the application of the blind method ensured the validity of this study.

Nevertheless, this study also has some limitations. Our study used the Rome III criteria to define functional constipation. New features in the Rome IV criteria for functional constipation may have a modest influence on clinical practice, though the majority of changes relative to Rome III are relatively minor for functional constipation^18^. Also, 90 patients who participated in the retest in this study were scheduled for surgery and were severely bothered by the symptoms of POP. The conclusion may not be extended to mild POP patients. Finally, the minimal clinically important change (MCID) of the CSS questionnaire was not included in our study, which was necessary when the CSS was used to evaluate the treatment effect.

## Conclusion

The Chinese version of the CSS questionnaire has great internal consistency, retest reliability, and structural validity. It could be commonly applied in clinical work and scientific studies in the evaluation of constipation symptoms in POP patients. The cut-off value for diagnosing functional constipation was 4.

## Supplementary Information

Supplementary Information.

## Data Availability

If the requirements are reasonable, the corresponding author can provide the data set generated and analyzed in the current research.
