# Supplementary material for: Evaluation of the Chinese version of the constipation scoring system in Chinese women with pelvic organ prolapse
Source: Sci Rep. 2022 May 5;12:7320. doi: 10.1038/s41598-022-11312-7 (PMC9072326; doi:10.1038/s41598-022-11312-7)
Supplement: Supplementary file 1 — Supplementary Information. [file 41598_2022_11312_MOESM1_ESM.pdf]

Evaluation of the Chinese version of the constipation scoring system in Chinese women with pelvic organ prolapse

Yanhua Liu[1], Man Tan[2], Cheng Tan[1], Xin Yang [1*]

[1]Department of Gynaecology, Peking University People's Hospital, Beijing, China

[2]Department of Gynecological Pelvic Floor and Oncology, Chongqing Health Center for Women and Children, Chongqing, China

Corresponding author: Xin Yang, Peking University People's Hospital, 11 Xizhimen South Street, Xicheng District, Beijing, China, xinyang_2003@sina.com

Supplementary Table 1 Structure validity of confirmatory factor analysis

Suitability test indicator	Ideal standard	Model result	Whether it meets the standard
CMIN/DF	< 3.00	1.064	conform
RMSEA value	< 0.08	0.021	conform
IFI value	> 0.90	0.992	conform
CFI value	> 0.90	0.992	conform
TLI value	> 0.90	0.988	conform
PNFI value	> 0.50	0.601	conform
PCFI value	> 0.50	0.673	conform

Supplementary Table 2 Factor loading matrix of confirmatory factor analysis

			Estimate	AVE	CR
1. Defecate frequency	<---	F1	0.551		
2. Difficulties: painful bowel movements	<---	F1	0.715		
3. Empty: Incomplete emptying	<---	F1	0.537		
4. Pain: abdominal pain	<---	F1	0.307	0.2806	0.6891
5. Time: Try defecation time (minutes)	<---	F1	0.529		
7. Failure to defecate: The number of failed defecations per 24 hours	<---	F1	0.454		
6. Assisted defecation: Assisted type	<---	F2	0.271	0.4975	0.6013
8. History: Course of constipation (year)	<---	F2	0.960		

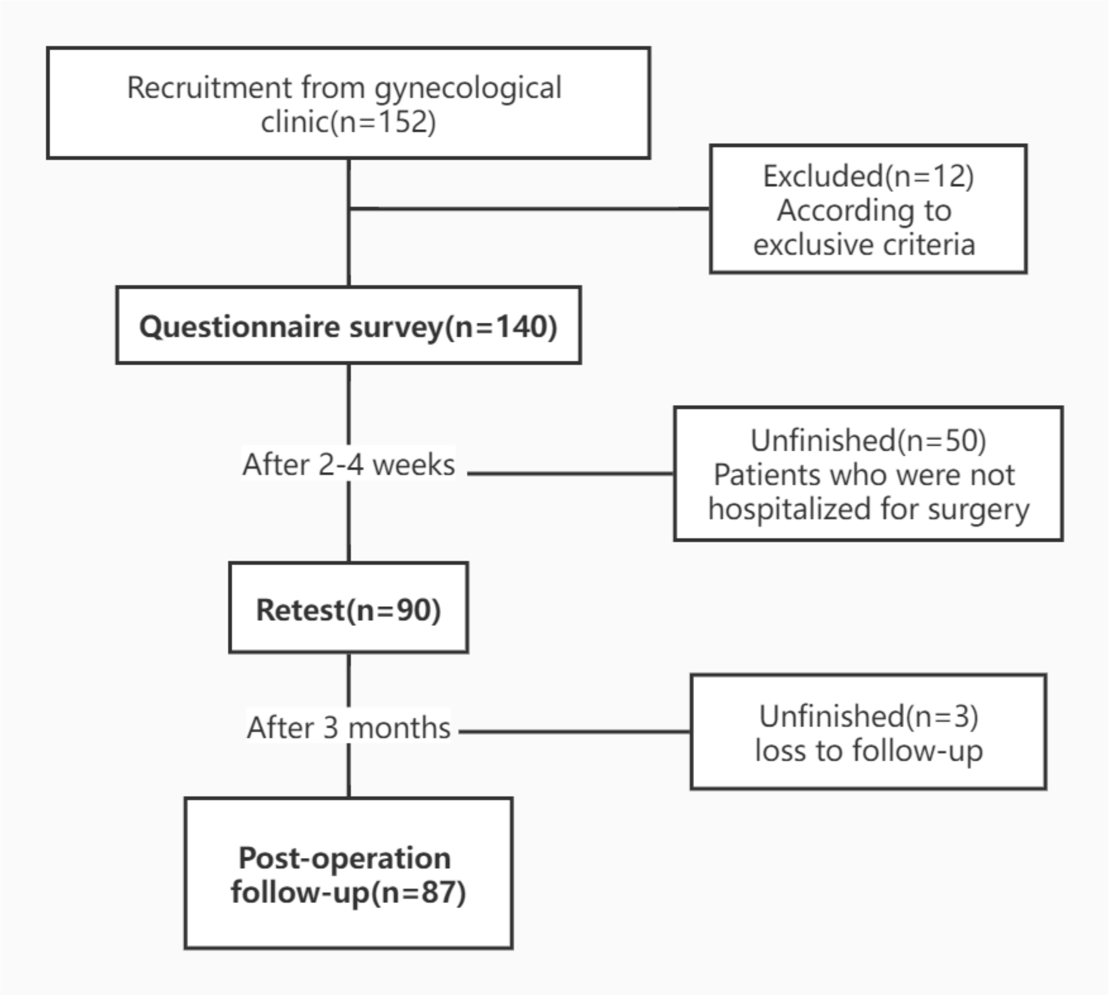

Supplementary Fig.1 The enrollment data
